# Spatial Transcriptomics as a Novel Approach to Redefine Electrical Stimulation Safety

**DOI:** 10.3389/fnins.2022.937923

**Published:** 2022-07-19

**Authors:** Quentin A. Whitsitt, Beomseo Koo, Mahmut Emin Celik, Blake M. Evans, James D. Weiland, Erin K. Purcell

**Affiliations:** ^1^Department of Biomedical Engineering, Institute for Quantitative Health Science and Engineering, Michigan State University, East Lansing, MI, United States; ^2^Department of Biomedical Engineering, Biointerfaces Institute, University of Michigan, Ann Arbor, MI, United States; ^3^Department of Electrical and Electronics Engineering, Gazi University, Ankara, Turkey; ^4^Department of Ophthalmology and Visual Sciences, University of Michigan, Ann Arbor, MI, United States

**Keywords:** microstimulation, spatial transcriptomics, safety, carbon fiber microelectrode (CFME), *in vivo* stimulation

## Abstract

Current standards for safe delivery of electrical stimulation to the central nervous system are based on foundational studies which examined post-mortem tissue for histological signs of damage. This set of observations and the subsequently proposed limits to safe stimulation, termed the “Shannon limits,” allow for a simple calculation (using charge per phase and charge density) to determine the intensity of electrical stimulation that can be delivered safely to brain tissue. In the three decades since the Shannon limits were reported, advances in molecular biology have allowed for more nuanced and detailed approaches to be used to expand current understanding of the physiological effects of stimulation. Here, we demonstrate the use of spatial transcriptomics (ST) in an exploratory investigation to assess the biological response to electrical stimulation in the brain. Electrical stimulation was delivered to the rat visual cortex with either acute or chronic electrode implantation procedures. To explore the influence of device type and stimulation parameters, we used carbon fiber ultramicroelectrode arrays (7 μm diameter) and microwire electrode arrays (50 μm diameter) delivering charge and charge density levels selected above and below reported tissue damage thresholds (range: 2–20 nC, 0.1–1 mC/cm^2^). Spatial transcriptomics was performed using Visium Spatial Gene Expression Slides (10x Genomics, Pleasanton, CA, United States), which enabled simultaneous immunohistochemistry and ST to directly compare traditional histological metrics to transcriptional profiles within each tissue sample. Our data give a first look at unique spatial patterns of gene expression that are related to cellular processes including inflammation, cell cycle progression, and neuronal plasticity. At the acute timepoint, an increase in inflammatory and plasticity related genes was observed surrounding a stimulating electrode compared to a craniotomy control. At the chronic timepoint, an increase in inflammatory and cell cycle progression related genes was observed both in the stimulating vs. non-stimulating microwire electrode comparison and in the stimulating microwire vs. carbon fiber comparison. Using the spatial aspect of this method as well as the within-sample link to traditional metrics of tissue damage, we demonstrate how these data may be analyzed and used to generate new hypotheses and inform safety standards for stimulation in cortex.

## Introduction

Rapid growth in the development of neural interface technology has outpaced a clear understanding of the effects of electrical stimulation on brain tissue as well as the underlying mechanisms of therapy. The Shannon equation, which defines a tissue damage threshold based on the intensity of electrical stimulation delivered (Shannon, 1992), was initialized from seminal studies relating signs of gross histopathological damage to the charge density and charge per phase of a single stimulus pulse. While the Shannon equation is practically useful, it has limitations: it provides a binary “damage/no-damage” outcome, without identifying signs of cellular stress that cannot be observed in histology. In these studies, Nissl or hematoxylin and eosin staining were used to reveal qualitative observations of neuronal shrinkage, hyperchromic cells, and vacuolization as signs of stimulation-induced damage (McCreery et al., 1990). However, cells may experience physiological changes due to electrical stimulation in the absence of clear signs of structural damage. Furthermore, the equation considers only characteristics of a single pulse (charge and charge density), but multiple studies have shown that pulse rate (McCreery et al., 1995; Gonzalez-Calle and Weiland, 2016) and duty cycle (McCreery et al., 2004, 2010) contribute to safety. Likewise, the Shannon limits may not be accurate for newer device designs, which feature cellular-scale electrode sizes (Cogan et al., 2016).

Spatial transcriptomics (ST) is an emerging technique which has the potential to reveal new biomarkers of microelectrode implantation and neurostimulation, providing a potentially powerful, more efficient way to identify positive or negative effects of stimulation. ST has been applied successfully to understand and evaluate biomarkers of pathology in neurodegenerative diseases (Chen et al., 2020; Marx, 2021). Our previous work surrounding recording electrodes implanted in motor cortex revealed hundreds of differentially expressed genes proximal to the implant site (Thompson et al., 2021; Whitsitt et al., 2021). When clustered into differently co-expressed gene modules, new effects associated with synaptic transmission, mitochondrial function, and metabolism were found at the interface of implanted recording electrodes (Thompson et al., 2021; Whitsitt et al., 2021; Moore et al., 2022). Simultaneously profiling all transcriptional changes–rather than pre-selecting a few markers of interest–can enable efficient unmasking of not only damage-associated effects, but also more nuanced and potentially neuroprotective effects of stimulation in the same tissue sample.

Here, we explored the use of ST to expand the current understanding of electrical stimulation on surrounding brain tissue. As intracortical microstimulation (ICMS) is a promising approach for the restoration of sensory information, we focused on the transcriptomic effects of ICMS in the visual cortex to establish a proof-of-principle. Our results illustrate several important phenomena motivating the use of the technique: (1) changes in the spatial pattern of individual gene expression in response to stimulation can be detected in a high-throughput manner [thousands of detected genes in each sample can be assessed for differential expression (DE)]; (2) the enrichment of *groups* of genes with known association to biological effects of interest in response to stimulation can be detected (for example, modules of genes associated with cell death, activity, or plasticity); (3) comparison between quantitative immunohistochemistry (IHC) results to gene expression profiles in the same tissue samples can be made, which allows direct benchmarking of new results to previous techniques; (4) pilot data suggest that both device design and stimulation parameters may influence ST results; and (5) gene expression is more sensitive than histology, revealing effects not immediately evident in immunofluorescence images. Our observations support the use of newer approaches in molecular biology to evaluate ICMS effects on surrounding brain tissue.

## Materials and Methods

### Electrodes and Stimulation Parameters

We conducted pilot experiments to investigate differential gene expression based on electrode array type, charge, and charge density. One array type was the Microprobes Microwire Array (MWA) (Microprobes for Life Science), with five 50-micron diameter Platinum-Iridium (PtIr) wires (∼2000 μm^2^ geometric area) and spaced at 300 μm. The other array was High-Density Carbon Fiber array (HDCF) (Patel et al., 2020) with five 6.8 μm diameter carbon fibers (CF) attached to silicon shanks and a percutaneous omnetics connector. A blow torch sharpened the CF into a cone shaped tip and removed approximately 140 μm of parlyene insulation from the tip (Welle et al., 2021), yielding a surface area of approximately 1500 μm^2^. All electrodes of both arrays were electroplated with PtIr (Lee et al., 2018; Valle della et al., 2021) to improve charge carrying capacity. Integrity of PtIr coating for all electrodes was verified with reduced electrochemical impedance spectroscopy (EIS) impedance values and altered cyclic-voltammetry readings. All stimulations were set as cathodic-first biphasic pulses at 200 μs per phase, with 5 μs interphase gap, and 50 Hz with varying current amplitude depending on chosen charge/charge density levels.

### Acute Implantation of Electrodes and Stimulation

Initial experiments tested early gene expression changes following electrical stimulation in comparison to a craniotomy control. While under isoflurane anesthesia, a single male Long-Evans rat was implanted with a HDCF array, stimulated for 1 h, and euthanized for tissue collection 2 h later. Stimulation parameters for the acute experiments were set at 25 μA with 200 μs per phase and 50 Hz, giving an estimated charge density of 0.347 mC/cm^2^ and charge per phase of 5 nC. Acute surgical procedures followed standard approaches previously detailed in Valle della et al. (2021). Briefly, a craniotomy was created over rat V1 cortex (−6.5 mm Anterior–Posterior, −3.5 mm Medial-Lateral, from Bregma), and a HDCF was inserted to a ∼600 μm depth from the cortical surface. The electrode was stimulated for 1 h, recording voltage transients (VTs) during stimulation to verify electrical contact via the brain-electrode interface. Two hours after the end of stimulation, the rat was deeply anesthetized with an overdose of sodium pentobarbital, decapitated, and the brain was removed and flash frozen using liquid nitrogen. All acute procedures were reviewed and approved by the Animal Care and Use Committee at Michigan State University.

### Chronic Implantation of Electrodes and Stimulation

Five Long-Evans rats of 250–300 g were each implanted with one MWA and one HDCF in each V1 hemisphere. Arrays were implanted in V1 (−6.5 mm Anterior–Posterior, −3.5 mm Medial-Lateral, from Bregma), using a 1.5 × 1.5 mm craniotomy and standard techniques to implant and secure the arrays (Welle et al., 2020). All electrodes were sterilized in a 48-h ethylene oxide cycle. The animals were implanted for 4 weeks to align with the terminal chronic time point assessed in previous literature (Biran et al., 2005). Of note, the underlying tissue response remains an important variable to consider in future studies evaluating the biological effects of electrical stimulation. Chronic animal implant procedures were reviewed and approved by the Animal Care and Use Committee at the University of Michigan.

The electrode with the smallest 1 kHz impedance at week 3 of implantation of each array was used for stimulation. Of the 10 arrays implanted, seven remained viable after 4 weeks, while three arrays were lost to head cap/connector loosening or open circuit. EIS was recorded under 1–3% isoflurane anesthesia before and after stimulation. The animal was connected to the PlexStim system (Plexon Inc., Dallas, TX, United States) and was placed in a clean cage with a small opening at the top, the connection tether was suspended overhead to avoid tangling with the animal or inducing stress on the animal’s head. After the animal recovered from isoflurane anesthesia, each electrode was stimulated for 7 h while the rat was awake and freely moving. Stimulus settings were defined as weak (2 nC, 0.1 mC/cm^2^ for MWA and 2 nC, 0.13 mC/cm^2^ for HDCF) or strong stimulation (20 nC, 1 mC/cm^2^ for MWA and 14.4 nC, 1 mC/cm^2^ for HDCF). These were chosen to be on either side of the 4 nC limit proposed by Cogan et al. (2016), as a rule-of-thumb limit for safe ICMS. Pulses were symmetric, cathodic-first, biphasic, 0.2 ms/phase, and 50 Hz. Using the Voltage Monitor and Current Monitor signals on PlexStim, VTs and current feedback were recorded during stimulation to monitor for compliant stimulation voltage and severe hydrolysis induction (both of which are indicated by distorted VT and current feedback waveforms). The PlexStim system was connected to the Tektronix TBS1032B oscilloscope (Tektronix, United States), and the resulting voltage and current readouts were saved as a comma-separated variable file for analysis in MATLAB. One day after stimulation, animals were deeply anesthetized with ketamine/xylazine cocktail and decapitated after sufficient muted hind-limb pinch response. As for acute samples, tissue was embedded in OCT and flash frozen in a bath of isopentane cooled with liquid nitrogen in accordance with previous reports (Chen et al., 2020; Whitsitt et al., 2021).

### Spatial Transcriptomics and Quantitative Immunohistochemistry

The frozen, whole brains were cryosectioned transversely at a depth of 500–600 μm with a 10 μm slice thickness to capture the electrode tip. Depth estimations were made based on the medial-lateral axis. The anterior–posterior axis is difficult to establish; therefore, there is some variation in the shape of the tissue sections. Sections were then mounted within a single 6.5 × 6.5 mm capture area on the Visium slide per tissue section. Two tissue sections were taken from the acute stimulation animal, and one tissue section was collected from each craniotomy control and chronic experiment animal. Immediately prior to immunohistological staining, tissue sections were fixed in chilled methanol for 30 min. IHC was performed using primary antibodies for neuronal nuclei (NeuN) at a concentration of 1:100 (Rb pAB to NeuN, Abcam, Cambridge, MA, United States, Cat#: 104225) and glial fibrillary acidic protein (GFAP) at a concentration of 1:400 (Monoclonal Anti-GFAP antibody. Millipore Sigma, St. Louis, MO, United States, Cat #: G3893-100). The secondary antibodies used were Alexa Fluor 488 (Anti-rabbit IgG, Invitrogen, Eugene, OR, United States, Cat #: A11034) for conjugation to the NeuN primary antibody and Alexa Fluor 647 (Goat anti-Mouse IgG, Invitrogen, Eugene, OR, United States, Cat #: A21235) for conjugation to the GFAP primary antibody. Hoechst was also applied at a concentration of 1:1000 as a universal nuclei stain. A Nikon A1R confocal microscope with a motorized stage was used to capture a large, stitched image of each tissue section made up of an array of smaller 20× magnification images. Four samples which showed signs of infection or excessive tissue deformation were removed from further analysis. Implant site location was estimated through a combination of known lateral distance from midline (3.5 mm) as well as the presence of prototypic markers of device-induced gene expression (e.g., *Ccl3*, *GFAP*). For quantitative IHC, custom MATLAB scripts were used to quantify neuronal densities (# of NeuN + nuclei/bin area) and the within-section normalized intensity of GFAP labeling within defined, binned distances from the implant site (Kozai et al., 2014a; Kucherenko et al., 2015; Salatino et al., 2019). GFAP intensity was measured in 10 μm bins, while neuronal density was measured in 100 μm bins. The quantified IHC data was used to compare to our newly defined stimulation-induced changes in gene expression.

After imaging the tissue sections, mRNA from each sample was released from the cells via a permeabilization enzyme (10x Genomics) applied for 18 min at 37°C. Polyadenylated (poly(A)) mRNA was bound to the spatially barcoded, poly(T) containing oligonucleotides on the Visium slide surface. A reverse transcription reaction extended each oligonucleotide with an anti-sense sequence of cDNA, from the bound mRNA strand. The original mRNA strand was then released from the slide via denaturation and the remaining oligonucleotide/cDNA molecule was prepared for second strand synthesis by the addition of a primer, or “template switch oligonucleotide.” After priming, a fully formed cDNA strand (containing the mRNA sequence, spatial barcode, and unique molecular identifier) was created through second strand synthesis. These final cDNA samples from each capture area were then released from the slide via KOH denaturation and transferred to individual tubes. The amount of cDNA from each capture area was then measured using qPCR and the remaining cDNA was amplified using the number of cycles from qPCR required to achieve 25% of the peak fluorescence value. The amplified cDNA was then purified using a paramagnetic bead-based size selection reagent, SPRIselect (Beckman Coulter Inc., Brea, CA, United States).

Amplified and purified cDNA samples from each capture area was transferred to the University of Michigan Advanced Genomics core for library preparation and sequencing. cDNA quality was assessed using the Tapestation 2200 (Agilent) and subjected to library preparation following the manufacturer’s protocol (10x Genomics). Final library quality was assessed using the LabChip GX (PerkinElmer). Pooled libraries were subjected to paired-end sequencing according to the manufacturer’s protocol (Illumina NovaSeq 6000). Bcl2fastq2 Conversion Software (Illumina) was used to generate de-multiplexed Fastq files. The SpaceRanger Pipeline (10x Genomics, version 1.3.1.) was used to align Fastq reads to a *Rattus norvegicus* reference transcriptome (Rnor_6.0) and select for reads containing a unique molecular identifier, spatial barcode, and gene annotation. Images and Space Ranger were used to generate a file compatible with the Loupe Browser software (10x Genomics, version 6.0.0) where clusters of spots on the slide can be drawn in reference to the IHC image of the sample. Additionally, raw counts files for individual genes expressed at each “spot” were delivered alongside spatial barcodes, allowing additional analyses and modeling to be performed.

### Gene Expression Analysis

Quality of sequencing was measured using FastQC (version 0.11.7/Java version 1.8.0_162) and quality of transcriptome alignment and sequencing depth was measured using Space Ranger. Sequencing quality was assessed based on the mean number of reads per spot as a metric for sequencing depth, where at least 50,000 reads per spot is recommended to reach an adequate sequencing depth. In our three acute experiment samples, the mean reads per spot was 80,540 and for our three chronic experiment samples, the mean reads per spot was 61,886. Of note, the paired-end sequencing quality of read 1 for Visium samples appears diminished after the 28th base pair because this is where the poly(T) portion of the slide oligonucleotides is incorporated into the sequence. Since there is no diversity for the base caller to distinguish between each base addition, the quality scores for these bases artificially appear low. Space Ranger accounts for this by removing bases beyond the 28th base in read 1 sequences.

For group comparisons, samples were aggregated based on timepoint using SpaceRanger. Aggregation normalizes the number of reads in each sample to correct for differences in sequencing depth. This is done by first calculating the number of reads in each sample that were confidently mapped to the reference transcriptome. The algorithm then subsamples the reads from the capture areas with a higher number of reads confidently mapped to equal the sample with the least number of confidently mapped reads. Once aggregated, the data are viewed in Loupe Browser where the number of sequencing reads in each spot, or clusters of spots, can be compared to one another to calculate log_2_ (fold change) (LFC). The two acute tissue sections that were stimulated were grouped into one cluster for calculation of LFC compared to the craniotomy control. Most tissue sections did not exhibit increased, localized GFAP protein expression, which is the traditional method for identifying the implant site. Therefore, the whole tissue sections were included in DE analysis. Spots without overlaying tissue in 100% of their area were removed from comparisons as well as spots under cryosectioning artifacts such as rolls, folds, and bubbles. DE was then calculated as the LFC between the average normalized counts for each gene in each condition. This second normalization step divides the counts for each gene in each spot by a size factor which is calculated as the total number of all counts in the spot divided by the average total number of counts in all spots used in the overall LFC calculation. The normalized counts of each gene in all spots in the cluster are then averaged and used to calculate the LFC between clusters. The resulting gene LFCs are the values reported in this article. Statistically significant DE genes were defined by a *p*-value > 0.05 and an LFC with an absolute value > 0.6. Due to the large size of each tissue section relative to the implant site, “low count” genes were included, which reports genes with an average number of reads per spot of <1. In order to extract biologically relevant information from the lists of DE genes, an open-source gene ontology (GO) tool (“GOrilla”) was used to assess enrichment of specific biological processes in the lists of significant DE genes from each comparison (Eden et al., 2009). Based on the presence of a significant number of genes associated with known processes in the lists of DE genes, the tool outputs biological processes possibly being activated or suppressed in the form of descriptive modules (“GO Terms”).

## Results

### Acute Experiment: Stimulation vs. Craniotomy

#### Immunohistochemistry and Differentially Expressed Genes

**FIGURE 1:**
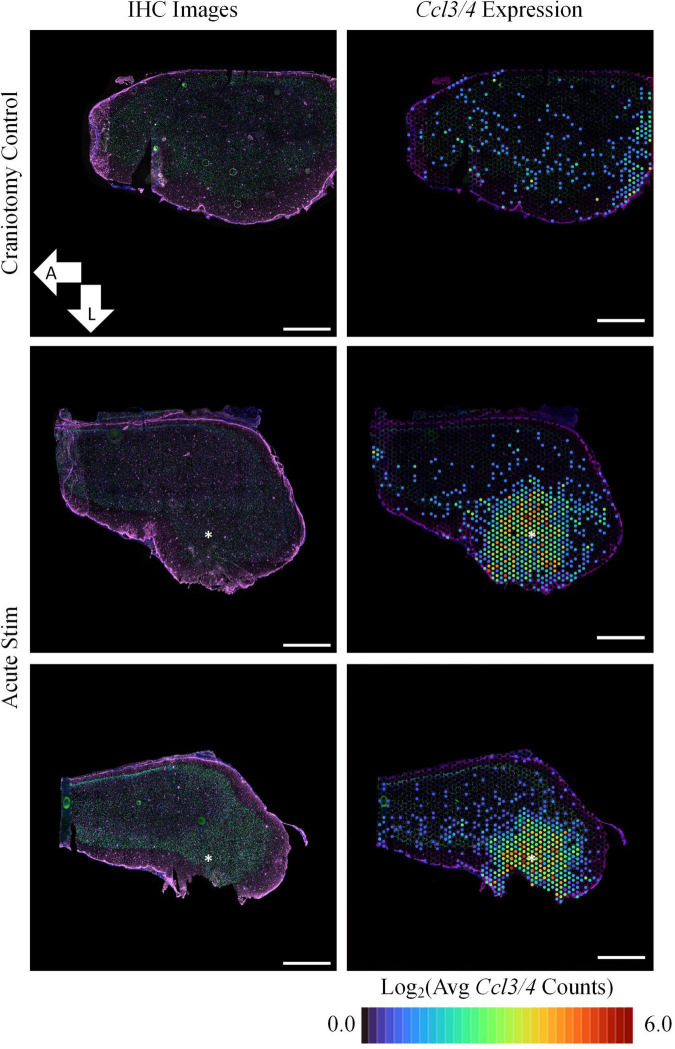
Acute experiments show upregulation of *Ccl3/4* following device implantation and stimulation. In comparison to tissue collected following a sham surgery (craniotomy only, top row), implantation of an HDCF electrode and stimulation was associated with strong upregulation of genes encoding inflammatory cytokines (*Ccl3/4*, right column). Immunohistochemistry (IHC) for astrocytes (GFAP, purple) and neuronal nuclei (NeuN, green) showed comparatively little effect on protein expression at the acute time point (nuclei are counterstained with Hoechst, blue). All images shown are transverse sections, with orientation as annotated. Right panels show the average spatial expression of *Ccl3* and *Ccl4* overlaid on the IHC image of each tissue section. Asterisks denote estimated implant site and arrows denote direction (L: lateral, A: anterior). Scale bars: 1.0 mm.

At the acute timepoint, 3 h after electrode insertion and initiation of electrical stimulation, IHC images showed no remarkable change in GFAP protein expression (Figure 1). Increased GFAP expression is a traditional method for locating electrode implants in *in vivo* histology and typically is observed 1 week after electrode insertion (Szarowski et al., 2003). Despite the lack of observable effects on GFAP protein expression, ST revealed that, compared to a craniotomy-only control, samples stimulated via HDCF differentially expressed 2,914 genes (*p*-value <0.05 and LFC magnitude >0.6). Among these DE genes, prominent upregulation of *Ccl3* (LFC: 3.18) and *Ccl4* (LFC: 3.12) was observed. These genes encode C-C Motif Chemokine Ligands 3 and 4, which act mainly as inflammatory cytokines. Increased *Ccl3/4* expression was localized with a radius of ∼1.0 mm in a region closely aligned with the electrode insertion coordinates. Additionally, *Ccl3/4* were not expressed in the craniotomy control. *Ccl3/4* were both present in related GO terms: “response to interleukin-1” (GO:0070555), “inflammatory response” (GO:0006954), and “response to cytokine” (GO:0034097). Increased expression of these genes in our data aligns with a previous study which reported upregulation of *Ccl2* and other chemokine-related genes surrounding intracortical electrode implants in the initial hours post-implantation (Bedell et al., 2020).

**FIGURE 2:**
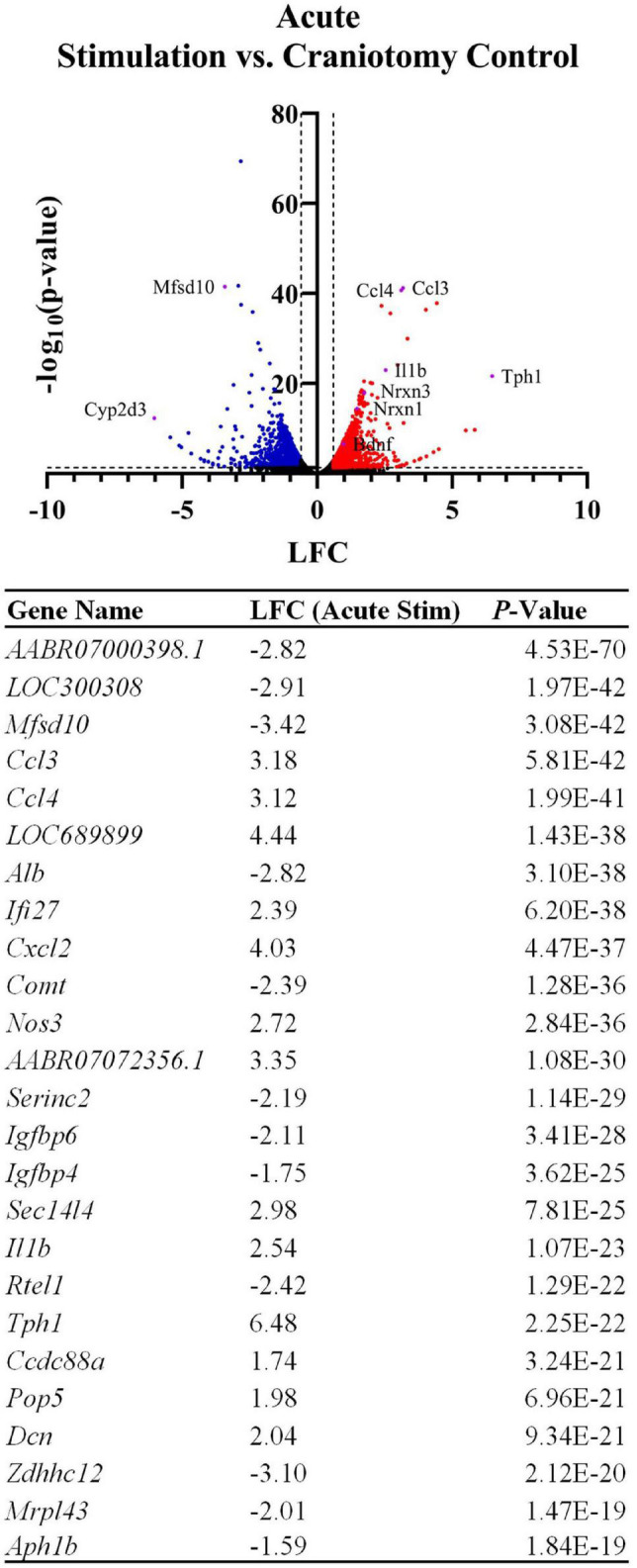
Device implantation and stimulation influences the expression of thousands of genes in comparison to a sham procedure (craniotomy only). The volcano plot and table show differentially expressed genes between the craniotomy control and the two acute stimulation samples, with genes related to inflammatory cytokines and related signaling cascades upregulated by the device insertion and stimulation. LFCs are reported as the change in gene expression in the stimulated samples compared to the control samples. The table shows the top 25 most significant DE genes.

**FIGURE 3:**
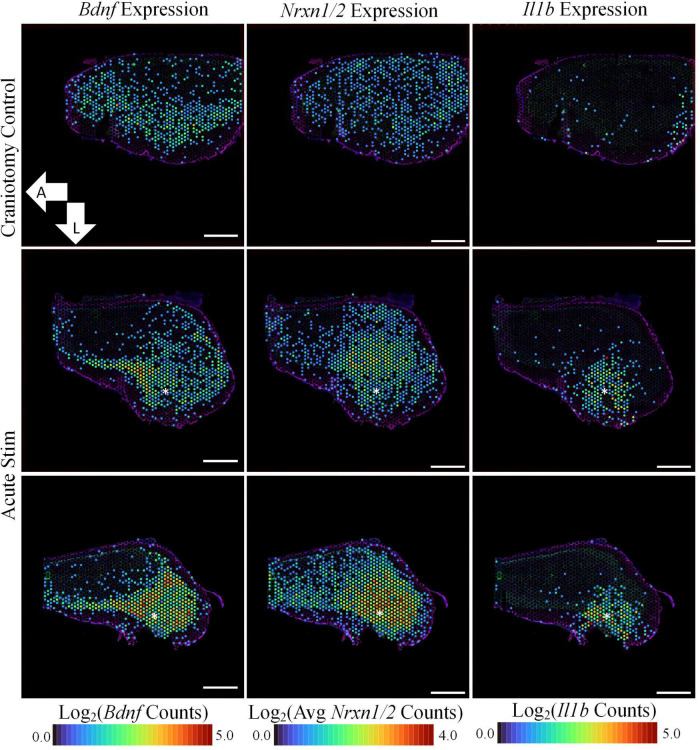
Acute stimulation is associated with upregulation of plasticity-associated genes. In addition to inflammation-associated genes, device insertion and stimulation caused the upregulation of genes associated with synaptic transmission. *Bdnf*, *Nrxn1*, and *Nrxn2* are all implicated in the formation and maintenance of synapses, and each were more prominently expressed in the stimulated samples in comparison to craniotomy control. Asterisks denote estimated implant site and arrows denote orientation (L: lateral, A: anterior). Scale bars: 1.0 mm.

Having confirmed expression of implant-associated genes, we searched the broader data set for additional effects. All DE genes are shown in the Figure 2 volcano plot, and the table highlights the top 25 most significant DE genes. The spatial profile of a subset of selected genes of interest are displayed in Figure 3. Similarly to *Ccl3/4*, interleukin-1 beta (*Il1b*) is a proinflammatory cytokine that was differentially expressed in the acute stimulation experiments (LFC: 2.54) and was listed in the same GO terms as *Ccl3/4*, further emphasizing the role of cytokine signaling as an effect of electrode implantation and stimulation.

Genes related to the viability of neurons and their synapses were also upregulated in the stimulated tissue sections. *Bdnf* was upregulated in stimulated samples (LFC:0.985, *p*-value: 2.73E-7) and was not observed in our non-stimulating electrode study or other non-stimulating transcriptomics studies in cortex (Bedell et al., 2020; Joseph et al., 2021; Thompson et al., 2021; Whitsitt et al., 2021). However, it has been reported in a transcriptomics study on stimulation in the dentate gyrus of mice (Pohodich et al., 2018). Additionally, Neurexin 1 (*Nrxn1*, LFC: 1.48, *p*-value: 3.73E-15) and Neurexin 3 (*Nrxn3*, LFC: 1.75, *p*-value: 1.04E-18) were found to be upregulated after stimulation compared to the craniotomy control. These genes encode proteins that are important for the formation of synapses (Zeng et al., 2007). GO analysis revealed GO terms associated with *Nrxn1/3* as “regulation of neurotransmitter levels” (GO:0001505) and “regulation of secretion” (GO:0051046), highlighting these genes’ roles in neurotransmission.

### Chronic Stimulation Effects

#### Voltage Transients

**FIGURE 4:**
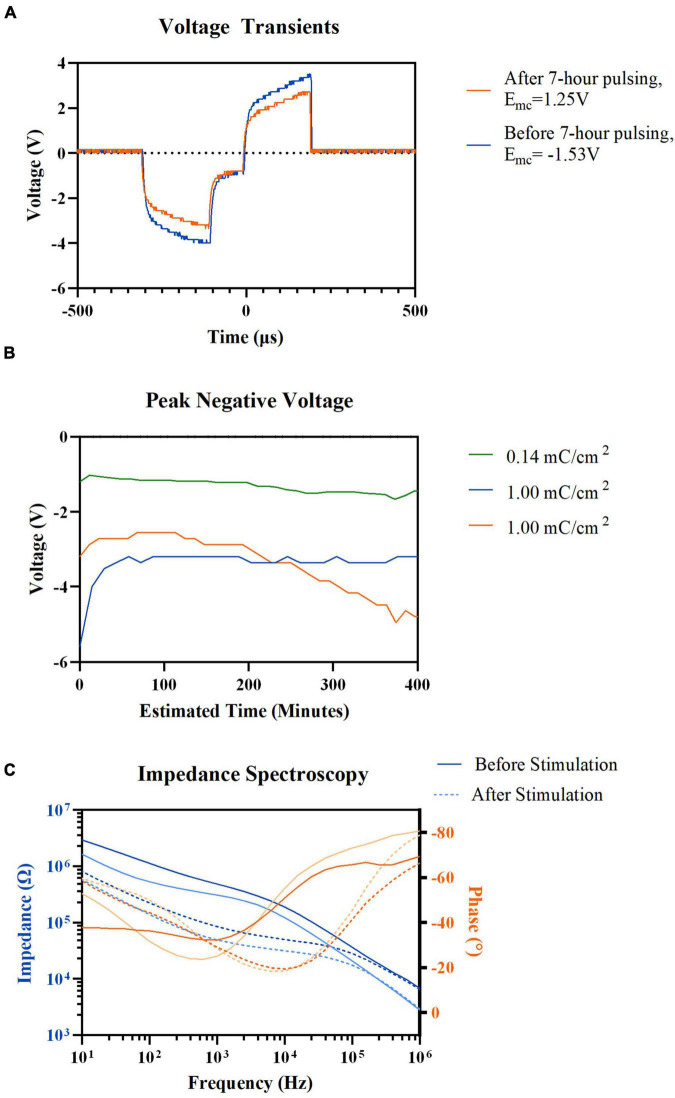
Electrochemical properties recording result before, after, and during 7-h electric stimulation. **(A)** Representative voltage transient of HDCF electrode before and after 7-h electric stimulation, 1.00 mC/cm^2^ current density, 72 μA. **(B)** Voltage transient peak negative values measured during cathodic phase of electric stimulation in three different HDCF electrodes: (green) 0.14mC/cm^2^, (blue) 1.00mC/cm^2^, and (orange) 1.00mC/cm^2^. **(C)** Pre-(Solid lines) and post-stimulation (Dotted lines) electrochemical impedance spectroscopy results of two HDCF electrodes (Blue) Impedance values and (Orange) Electrode frequency values.

Seven-hour pulsing of the HDCF arrays, even at high current density of 1 mC/cm^2^, showed no visible alteration to VT waveform (Figure 4A). The maximum negative potential excursion (E_*mc*_) was estimated before and after chronic stimulation and was observed to exceed the water window for platinum (−0.6 to 0.9 V) (Hudak et al., 2010). However, the transient waveforms still maintained the expected shape regardless of the high potential. The peak values of VTs were monitored (Figure 4B). Of the two electrodes stimulated with high current density of 1 mC/cm^2^, one exhibited an increasing peak voltage while the other maintained a stable peak voltage over the 7-h stimulation. Regardless of peak voltage increase, both carbon fibers stimulated with 1 mC/cm^2^ experienced a decrease in mid-to-low frequency impedance values (Figure 4C).

We did not strictly limit the applied current based on an electrochemical definition of injectable charge (Cogan, 2008). Studies have shown that *in vivo* charge levels, proven safe through histological analysis, exceeded electrochemically defined injectable charge (Leung et al., 2014). Instead, we monitored VT for distortions that indicate severe hydrolysis, which appear as flattening of the VT peaks. Such distortions were not detected. Microelectrodes, like those used in this study, may produce only small quantities of H_2_ and O_2_ when pulsed at high levels of charge and tissue may have the capability to buffer these by-products. Electrode impedance was decreased after stimulation, which is consistent with other reports when pulsing or application of DC voltage decreased impedance (Pudenz et al., 1975; Weiland and Anderson, 2000; Otto et al., 2006).

#### Comparison of Quantitative Immunohistochemistry and Spatial Gene Expression Patterns

**FIGURE 5:**
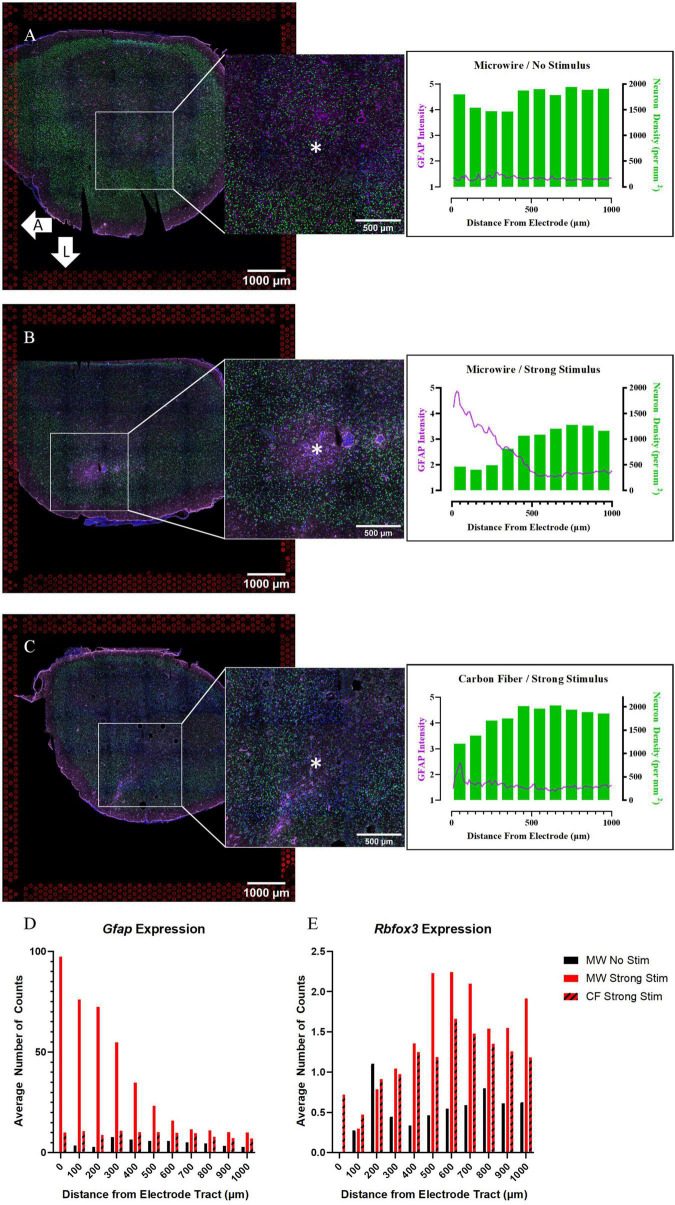
Quantitative immunohistochemistry of chronically implanted samples indicates that both device type and stimulation delivery may influence traditional safety metrics (neuronal density, glial reactivity). **(A)** Tissue collected at the 4-week time point from a sample implanted with a MWA that delivered no stimulation shows relatively minimal local glial (GFAP) reactivity or neuronal loss (NeuN density). **(B)** Tissue collected from a sample implanted with a MWA that delivered electrical stimulation following 4 weeks of device implantation shows evidence of local gliosis and neuronal loss. **(C)** Tissue collected from a sample implanted with a HDCF that delivered electrical stimulation following 4 weeks of device implantation shows lesser evidence of local gliosis and neuronal loss in comparison to the MWA/Stimulation sample shown in panel **(B)**. **(D)** Spatial expression of *Gfap* counts at increasing distances from the electrode tract in all three chronic samples align with IHC results. **(E)** Spatial expression of the gene *Rbfox3*, which encodes the NeuN protein, at increasing distances from the electrode tract in all three chronic samples. An advantage of the spatial transcriptomics assay is that spatial profiles of gene expression can be compared directly with more traditional quantitative immunohistochemistry metrics. Asterisks denote estimated implant site and arrows denote orientation (L: lateral, A: anterior). Scale bars: 1.0 mm.

As in the acute experiments, we first assessed outcomes relative to traditional IHC for the chronic experiments as well. IHC images and their respective quantification for GFAP immunofluorescence and neuron density (based on NeuN) are shown in Figure 5. In the absence of stimulation, the MWA implant showed little to no GFAP reactivity or neuronal loss surrounding the expected implant site (Figure 5A). This is somewhat unexpected as microwire electrode implants are usually associated with increased GFAP immunoreactivity (Winslow and Tresco, 2010); however, this may be due to the variability in GFAP reactivity between electrode implants of the same type (Michelson et al., 2018) or variance due to cryosectioning depth accuracy. In comparison, the MWA that delivered electrical stimulation shows pronounced GFAP expression and neuronal loss surrounding the implant (Figure 5B). HDCF with strong stimulation experienced no localized GFAP immunofluorescence proximal to the stimulation site as measured by quantitative IHC; however, this tissue section qualitatively exhibited a possible recruitment of astrocytes from the cortex periphery (Figure 5C). Neuronal density was decreased near both MWA and HDCF stimulation sites, indicating a possible separation of astroglial reactivity and neuronal loss pathways between the two electrode designs.

To directly compare the spatial distribution of transcriptional profiles to IHC, we plotted counts of selected genes as a function of distance from the electrode location. In Figure 4 and Supplementary Figure 1, the spatial expression of genes is binned in distance increments from the device location in the same manner as in the quantitative IHC analysis. Figure 5D shows the spatial pattern of *Gfap* expression in the chronic experiment samples is similar to the spatial pattern of GFAP intensity in the IHC images. The stimulated MWA sample had the largest peak in *Gfap* expression close to the electrode tract, with a steady decrease in expression until it plateaus at approximately 700 μm. On the other hand, *Gfap* expression in both the stimulated HDCF and non-stimulated MWA displayed no localized upregulation, although the increase in baseline *Gfap* expression in the stimulated HDCF was noted in comparison to the stimulated MWA. *Rbfox3* encodes the NeuN protein and followed a similar pattern to neuronal density in the two stimulus conditions, with a decrease near the electrode tract and a steady increase as distance from the electrode tract increases. An important note about *Rbfox3* expression is that expression levels are relatively low (the max average number of counts is around 2), which is likely why the first bin, containing only one “spot,” has 0 counts of *Rbfox* for both microwire conditions. These spatial measurements of gene expression exhibit the ability of this ST method to directly compare gene expression to current benchmarking methods used to establish tissue response severity, within the same sample tissue section.

#### Microwire vs. Carbon Fiber Strong Stimulation

**FIGURE 6:**
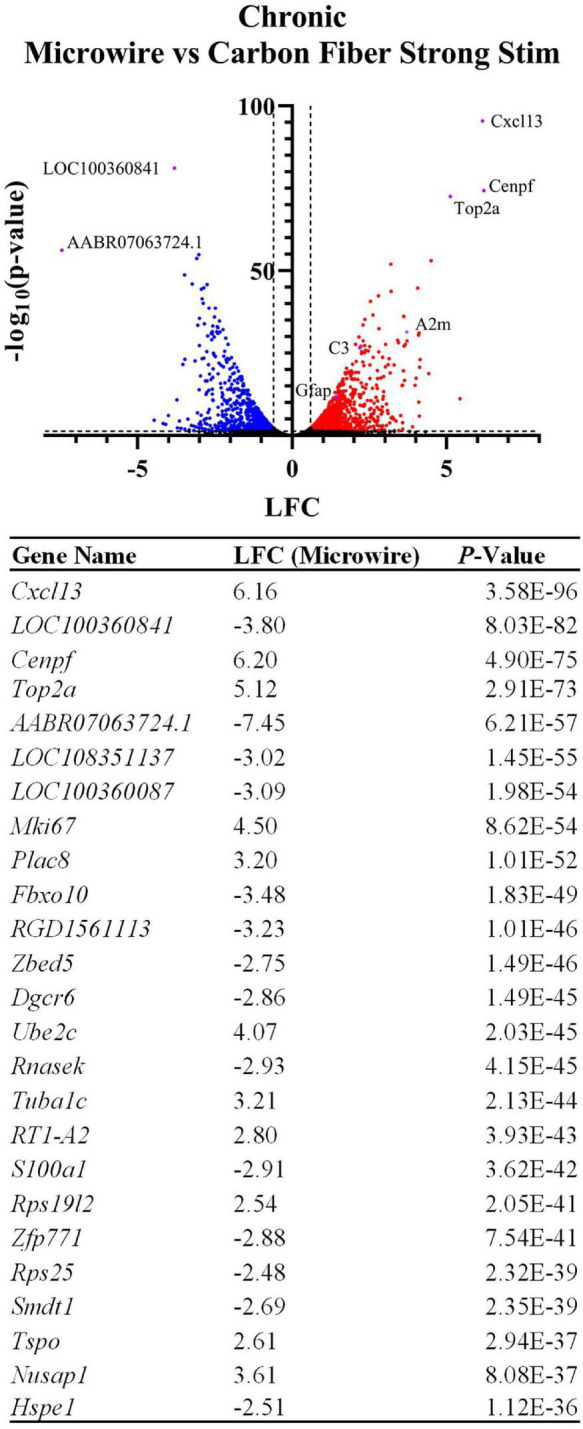
Device type influences the expression of thousands of genes following chronic implantation and electrical stimulation. The volcano plot and table show differentially expressed genes between the two device types delivering a strong electrical stimulation (LFCs indicate differentially expressed genes in MWA relative to HDCF). Genes related to inflammation and cell cycle entry were preferentially upregulated by stimulation delivered by the MWA. The table shows the top 25 most significant DE genes.

**FIGURE 7:**
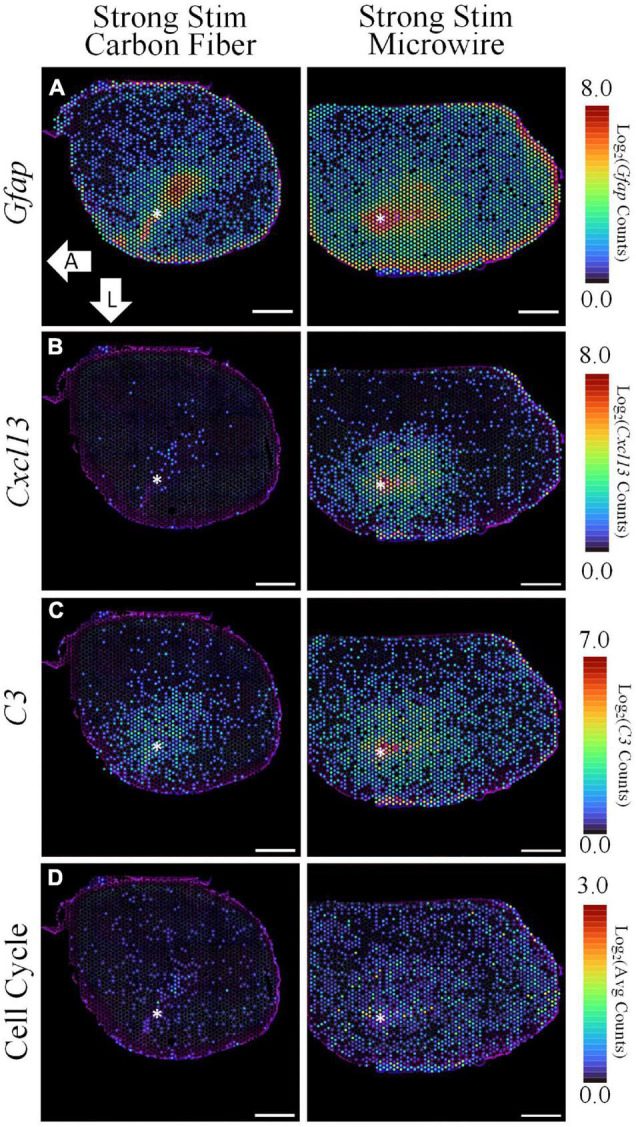
The spatial pattern of selected differentially expressed genes illustrate the upregulation of inflammation and cell cycle-associated pathways following electrical stimulation delivered by a MWA in comparison to a HDCF. While *Gfap* is notably increased in both samples, stimulation delivered by the MWA is associated with genes included in a cytotoxic, “Cell-Killing” gene ontology term (*C3*, *Cxcl13*). A less prominent, but significant, upregulation of cell cycle-associated genes with the MWA-stimulation condition was an unexpected result revealed by the spatial transcriptomics assay. Average expression of DE genes making up the “Cell Cycle” GO Term is shown. Asterisks denote estimated implant site and arrows denote direction (L: lateral, A: anterior). Scale bars: 1.0 mm.

To explore potential device-dependent effects, DE analysis was performed to compare the microwire stimulus condition to the carbon fiber stimulus condition. All differential gene expression from this comparison is reported as the LFC in the stimulated MWA relative to the stimulated HDCF. DE analysis between these two conditions resulted in 1,929 DE genes with a *p*-value < 0.05 and a LFC magnitude >0.6 (Figure 6). Using *Gfap* as an initial assessment, both the MWA and HDCF show upregulation surrounding the expected implant site (Figure 7A); however, when the whole tissue sections are compared, *Gfap* is found to be more upregulated in the MWA condition (LFC: 1.44, *p*-value: 1.59E-12). This is likely due to the larger area of increased *Gfap* expression in the MWA condition compared to the HDCF condition. Prominently, *Cxcl13* is highly upregulated (LFC: 6.16, *p*-value: 3.58E-96); *Cxcl13* expression is clearly expressed in the MWA stimulus condition and almost non-existent in the carbon fiber stimulus condition (Figure 7B). *Cxcl13* is present in the GO term “B-cell chemotaxis” (GO:0035754) as well as in the GO terms “endothelial cell chemotaxis to fibroblast growth factor” (GO:0035768), “cell chemotaxis to fibroblast growth factor” (GO:0035768), and “B-cell chemotaxis across high endothelial venule” (GO:0035769). Taken together, these GO terms support the role for *Cxcl13* as a chemoattractant for B lymphocytes across the blood–brain barrier. *C3* is also upregulated in the MWA condition (LFC: 2.19, *p*-value, 2.25E-27, Figure 7C). This finding is an important validation of this method, as *C3* has been detected surrounding chronic electrodes previously (Joseph et al., 2021; Thompson et al., 2021). C3 is a well-known initiator of the complement cascade which induces inflammation and has been found to be produced by reactive astrocytes (Liddelow et al., 2017; Hasel et al., 2021). Taken together, these observations suggest a more pronounced induction of inflammatory pathways following strong stimulation delivered by the MWA in comparison to the HDCF.

GO analysis for the microwire vs. carbon fiber stimulation comparison yielded mainly terms related to cell cycle progression, which was an unexpected finding that will need further investigation. For visualization, the average expression of upregulated DE genes in the “positive regulation of mitotic nuclear division” (GO:0045840) and “cell cycle” (GO:0007049) GO terms are shown in Figure 7D (*Cenpf*, *Tuba11c*, *Ccnb1*, *Cdk1*, *Mki67*, *Cnpe*, *Ube2c*, *Nusap1*, and *Aurkb*). While cell cycle re-entry has been proposed previously as a mechanism underlying glial reactivity and neuronal loss surrounding implanted electrodes (Purcell et al., 2009), the possibility of stimulation-evoked transition from a quiescent to proliferative state is a unique observation in this ST data set.

#### Microwire Strong Stimulation vs. No Stimulation

**FIGURE 8:**
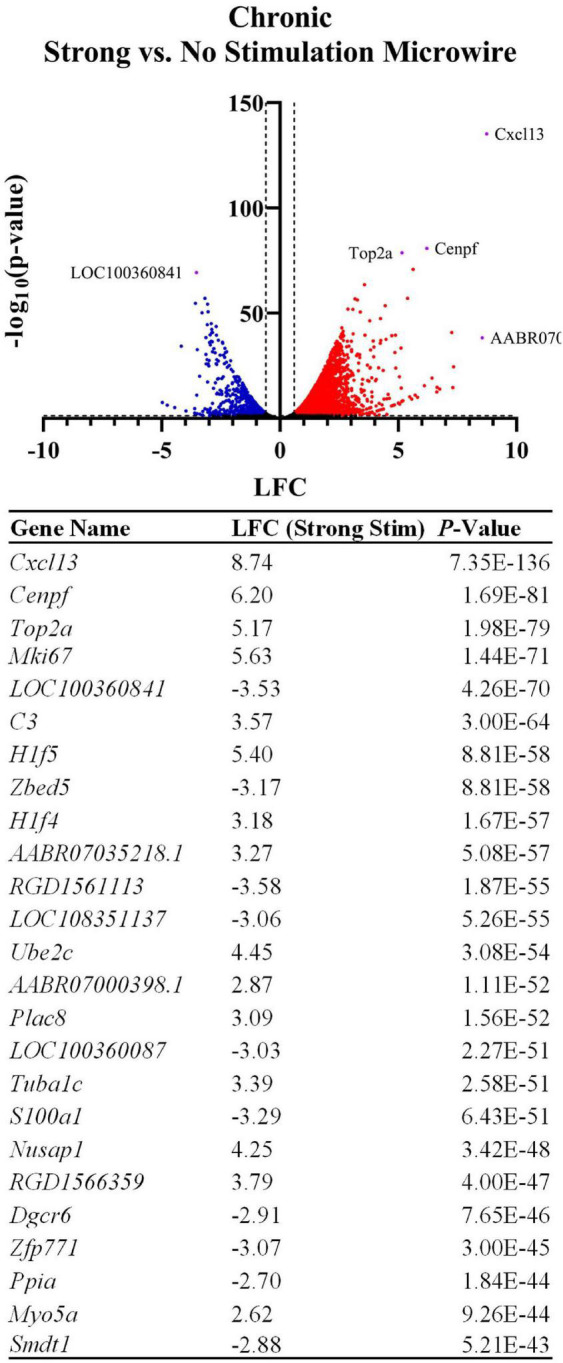
Stimulation influences the expression of thousands of genes following chronic implantation. The volcano plot and table show differentially expressed genes between an MWA delivering a strong electrical stimulation vs. no stimulation (LFCs indicate differentially expressed genes in strong stimulation relative to no stimulation). Similarly to the MWA vs. HDCF strong stimulation comparison, inflammation-associated genes were upregulated by the MWA delivering stimulation. However, the associated LFCs were relatively more pronounced when referenced to the MWA non-stimulated sample (for example, *Cxcl13* shows a LFC = 8.74, vs. LFC = 6.16 in Figure 6). The table shows the top 25 most significant DE genes.

**FIGURE 9:**
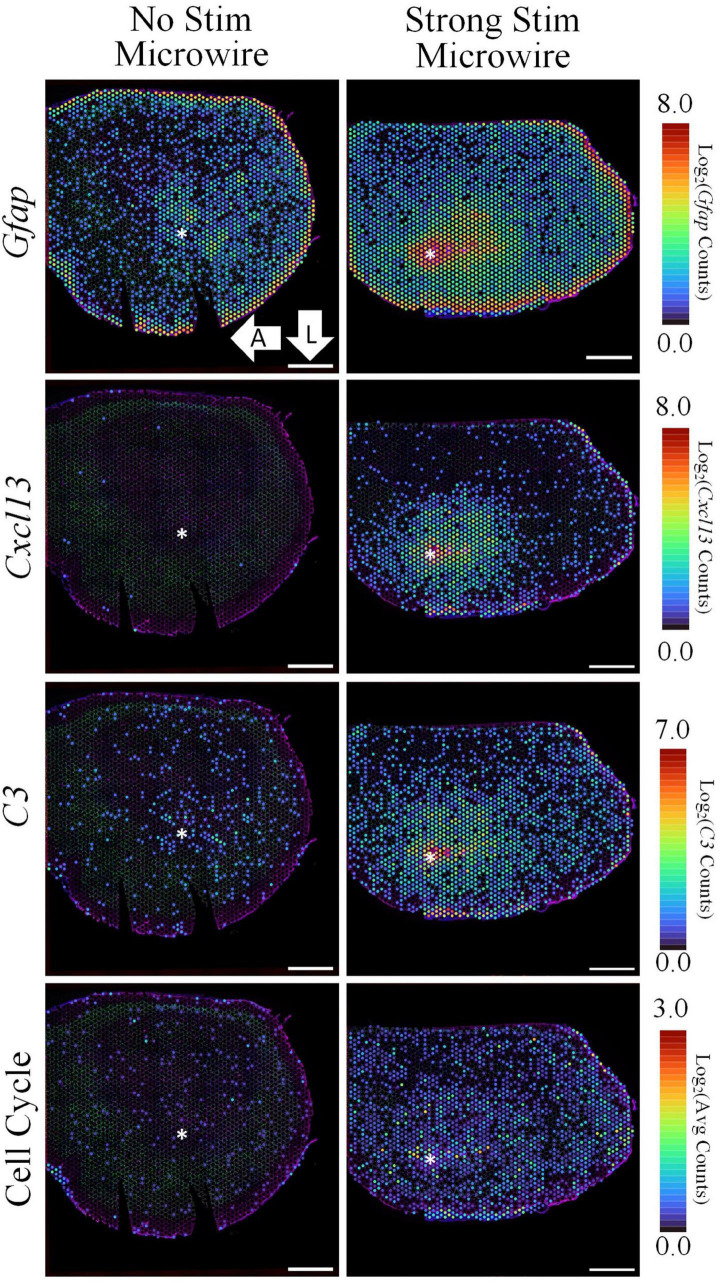
The spatial pattern of selected DE genes illustrate the upregulation of inflammation and cell cycle-associated pathways following electrical stimulation delivered by a MWA in comparison to no stimulation. Stimulation delivered by the MWA is associated with increased expression of genes included in a cytotoxic, “Cell-Killing” gene ontology term (*C3*, *Cxcl13*). Upregulation of cell cycle-associated genes with the MWA-stimulation condition was an unexpected result revealed by the spatial transcriptomics assay. Average expression of DE genes making up the “Cell Cycle” GO Term. Asterisks denote estimated implant site and arrows denote direction (L: lateral, A: anterior). Scale bars: 1.0 mm.

A DE comparison between the stimulated and non-stimulated MWAs was then used to observe changes in gene expression between stimulated vs. non-stimulated tissue. LFCs are reported as the change in gene expression in the stimulated sample compared to the non-stimulated sample. DE analysis resulted in 3,792 DE genes between these two conditions with a *p*-value < 0.05 and a magnitude LFC > 0.6. All measured genes are shown in the volcano plot in Figure 8 and the top 25 most significant DE genes are shown in the table. Using *Gfap* as an initial assessment, only the stimulus condition shows a strong upregulation around the electrode tract (LFC: 1.74, *p*-value: 4.46E-19, Figure 9A). Overall, the list of DE genes in this comparison is very similar to the DE in the microwire vs. carbon fiber stimulus comparison. *Cxcl13* is also DE in this comparison (LFC: 8.74, *p*-value: 7.35E-136, Figure 9B), as is *C3* (LFC: 3.57, *p*-value: 3.00E-64, Figure 9C); however, the LFCs are more pronounced in this comparison. This observation suggests a gradient of inflammation/immune response gene expression, where the stimulated MWA shows the highest expression of these genes, followed by the stimulated HDCF, and the least amount of expression in the non-stimulated MWA. GO analysis of DE genes in the microwire stimulus vs. no stimulus comparison revealed a majority of GO terms relate to cell cycle progression (Figure 9D).

## Discussion

Spatial transcriptomics is a recently developed approach which can reveal the activation of signaling pathways related to cellular stress, death, activity, plasticity, and metabolism in combination with traditional immunohistological staining techniques. While our observations are preliminary in nature due to a limited sample size, the proof-of-concept data support the idea that ST can be used to reveal nuanced biological effects of stimulation. ST could, in turn, influence the definition of “safe” stimulation and add insight into the mechanisms of the therapeutic effects of neuromodulation. In support of the validity of the method, the data obtained in this study are consistent with previously reported effects of electrode implantation and electrical stimulation. The prominent increase in chemokine expression in the hours following insertion, the induction of *Gfap* at chronic time points, and the increase in *Bdnf* expression following stimulation all align with previously reported effects (Pohodich et al., 2018; Bedell et al., 2020; Joseph et al., 2021).

The high-throughput nature of ST contextualizes these observations by detecting the expression of related genes, as well as benchmarking results with quantitative IHC. For example, important cytokines upregulated in the acute stimulation samples included *Il1b*, C-X-C motif chemokine ligand 2 (*Cxcl2*), and interferon alpha inducible protein 27 (*Ifi27*). *Ccl3* and *Ccl4* were upregulated in the acute experiments and encode pro-inflammatory cytokines in the macrophage inflammatory protein-1 (MIP-1) family. These signaling proteins act on CC chemokine receptors expressed by lymphocytes and macrophages and have been linked to a wide array of physiological effects including the migration of these cells across the blood–brain barrier (Takeshita and Ransohoff, 2012). The expression of *Ccl3* and *Ccl4* can be increased through activation of immune cells by interleukin-1 beta (IL-1β) (Maurer and von Stebut, 2004), which is also transcriptionally upregulated in these acutely stimulated samples. Furthermore, *Il1b*, *Ccl3*, and *Ccl4*, as well as other cytokine genes such as *Cxcl2* and *Ifi27* found DE at this early timepoint, trigger mitogen-activated protein kinase (MAPK) cascades that activate transcription factors. *Ccl3* and *Ccl4* are both listed in the GO Term “positive regulation of ERK1 and ERK2 cascade” (GO:0070374). Activation of the ERK1/2 pathways is also interesting in the context of increased *Bdnf* expression since these signaling pathways are necessary for BDNF-driven dendrite spine development in hippocampal pyramidal neurons (Alonso et al., 2004). The observations collected in acute-stimulated samples suggest the co-activation of a combination of inflammatory and neuroprotective pathways. Our previous study using non-stimulating Michigan-style electrode arrays in motor cortex did not find an upregulation in *Bdnf* or *Nrxn1/3* at any timepoint; however, we did find a downregulation of *Nrxn3* at 24 h and 1 week post-implantation (Whitsitt et al., 2021). Although it is difficult to establish a causal relationship from this study or in comparison to our previous study, these findings suggest that the inflammatory and neuroprotective pathways are being activated independently, and that the neuroprotective pathway is driven by cortical stimulation.

Analysis of chronic samples highlighted an important benefit of the approach: the ability to compare transcriptional results directly with quantitative IHC. Quantification of GFAP and NeuN staining are commonly used to assess electrode-related damage and stimulation-induced effects (Butson et al., 2006; McCreery et al., 2010). Comparing the GFAP IHC results to the spatial expression of *Gfap* revealed remarkable similarity, with the exception that spatial expression of *Gfap* gene expression extends far past the distance of increased GFAP protein expression measured with IHC. This finding indicates changes in pro-inflammatory gene expression are more widespread than is measured using traditional IHC metrics. Likewise, quantitative IHC of NeuN densities aligned with the spatial expression of the NeuN-encoding gene (*Rbfox3*). When considering such spatial relationships between gene expression and protein expression, it is important to note that the mechanisms affecting mRNA translation into protein, and the target of the protein, are much more complex than a simple one-to-one relationship (Buccitelli and Selbach, 2020). Factors such as protein and mRNA turnover, protein transport, and cell proliferation can all confound interpretations of gene expression leading to protein expression. While we have been able to characterize the similarities and differences in *Gfap* and *Rbfox3* expression relative to their proteins’ expression in these samples, it will be useful to validate interpretations of other genes’ expression levels to their corresponding proteins using IHC or other quantitative, spatial methods in future work.

An additional important strength of this approach is the ability to assess condition-related effects using DE analysis. Gene expression between the MWA and HDCF stimulus conditions reveals an upregulation of many pro-inflammatory genes such as *Cxcl13* and *C3* in the stimulated MWA sample, but not the stimulated HDCF sample (Supplementary Figure 1). The spatial expression pattern of these genes, along with the stereotypical inflammatory biomarker *Gfap*, is increased near the MWA and then declines to baseline levels further away from the device. On the other hand, *Rbfox3* expression is decreased in both stimulated samples. This closely mimics the results obtained from traditional quantitative IHC analyses of GFAP and NeuN. These results would indicate that both device designs led to neuronal loss; however, the MWA created more inflammation leading to gliosis compared to the HDCF. Importantly, both stimulated samples (HDCF and MWA) received stimulation considered to be damaging by the Shannon equation. While preliminary, this observation supports the idea that other aspects of device architecture also may need to be considered when determining safety limits of intracortical stimulation. The HDCF array is more likely to avoid the significant Iba1 and GFAP protein expression, voids left in tissue, and kill zone surrounding the glial scarring found in typical microelectrode archetypes such as the “Michigan” shank probe, microwire arrays, and “Utah” needle bed (Patel et al., 2016, 2020; Welle, 2021). The sub-neural cross-sectional area of individual carbon fibers of the HDCF resulted in reduced voids and subsequent kill zones. In addition, reduced surface area and subsequently the friction applied to the extracellular matrix lowered the insertion force required to insert into tissue. In this study, the non-stimulated vs. stimulated MWA comparison yielded many of the same DE genes as the MWA vs. HDCF comparisons, possibly indicating the prominence of electric stimulation as a driver of gene expression changes.

Perhaps most importantly, ST can move beyond neuronal loss and glial encapsulation to unmask specific signaling pathways (necrosis/apoptosis, plasticity, activity etc.). The upregulation of *Bdnf* and *Nrxn1/3* in the acute stimulation conditions is an important example of how ST may reveal unexpected results. *Bdnf* is a well-known neurotrophic factor that has been linked to neuronal health and synaptic plasticity (Alonso et al., 2004; Kajiya et al., 2008). *Nrxn* genes, which are also upregulated in the acute stimulation samples, are closely linked to synapse formation (Missler et al., 2003). This could indicate either synapse formation or repair after acute stimulation. This seems likely to be a result of the stimulation, given that *Nrxn3* is downregulated at 24 h and 1 week in our previous study, which assessed electrode implantation in the absence of an electrical stimulus (Whitsitt et al., 2021). Likewise, *Ccl3/4* overexpression in the acute stimulation experiments may play a role in ERK1/2 signaling pathways, and ERK signaling via *Ccl3/4* may stimulate neuronal growth and synapse formation via BDNF. Alternatively, ERK activation can initiate apoptosis (Cagnol and Chambard, 2010), which plays a reported role in neuronal cell loss surrounding electrode implants in the brain (Kozai et al., 2014b). The ST method can detect signatures of cell death in our samples, as evidenced by the association of the “cell-killing” GO-term with strong stimulation delivered by the MWA (*C3/Cxcl13*). In another example of the advantages of the technique, investigation of the data provided added insights into the nature of the inflammatory response to stimulating electrodes. Two branches of the immune response (innate and humoral) were identified in this study that were activated at separate timepoints. The innate immune response (macrophages) is possibly being driven by *Ccl3/4* expression in the acute phase, while the humoral immune response (B-cells) is possibly being driven by *Cxcl13* in the chronic phase. The combination of these observations support the ability of ST to provide more specific information on the signaling pathways activated by electrical stimulation than can be revealed by histology alone.

One important caveat to the current results is that there is a limited sample size in the study. As such, the specific findings from our comparisons should be interpreted with caution, because there is the possibility that subject-specific effects contributed to the results. Likewise, the exact localization of the implant location can be a challenge. Tissue sections in this study were taken 500–600 μm from the surface of the brain, centered on the implant site on the medial-lateral axis; however, the anterior–posterior orientation of the brain during cryosectioning may lead to differences in depth farther away from the implant site. We also chose to report “low count” genes here, which includes genes that have an expression level less than one read per spot. Since the large size of each tissue section relative to the electrode tract may mask highly localized and lowly expressed changes in gene expression, we chose to include this data. However, the DE genes reported may exhibit a large LFC with a small *p*-value but do not necessarily exhibit widespread expression. Additional methodological considerations to be explored in the future include the use of perfusion prior to tissue collection, re-orienting the sectioning plane to capture layer-specific effects, and the potential to infer the contributions of individual cell types to results through factorization and deconvolution strategies (Lee and Seung, 1999; Cobos et al., 2018, 2020). Finally, alternative assays have been used in other fields, as well as the description of the tissue response to non-recording electrodes, which could provide valuable insights. Some of these approaches offer improved spatial resolution, combined proteomics, perturbation in thick tissue sections, etc. (Bedell et al., 2020; Joseph et al., 2021; Zhang et al., 2021). While our approach has a benefit of directly comparing current histological methods with a deeper understanding of gene expression, it sits in a broader family of new techniques which could provide valuable insights into the impact of stimulation on brain tissue in the future.

## Conclusion

Our study showed changes in gene expression after both acute and chronic stimulation experimental paradigms. In the acute experiments, spatial expression patterns of pro-inflammatory genes were shown as well as the more unexpected results of *Bdnf* and *Nrxn1/3* upregulation after stimulation. In the chronic experiments, we explored spatial patterns of pro-inflammatory genes *Cxcl13* and *C3* and revealed widespread upregulation of genes relating to cell cycle progression. Comparisons were drawn in the chronic experiments to reveal differences between stimulated and non-stimulated tissue as well as differences in gene expression based on device design. We also used traditional quantitative IHC analysis to benchmark our findings with a method commonly applied to measure damage from electrode insertion and stimulation. Transcriptomics, in a general sense, can provide a variety of benefits to our current understanding of stimulation-related damage and safety, which are evident in our data. This technique also allows us to measure transcriptional changes without histological effects, such as the absence of GFAP expression in the acute experiments. We also are able to describe gradients of pro-inflammatory genes using the quantitative aspect of RNA-sequencing to provide more nuance in differences between conditions. Finally, we have demonstrated the ability of this technique to provide spatial context to transcriptional changes following electrical stimulation in the brain. In future experiments, ST may be important for understanding interaction of stimulation effects with multi-tined electrodes, layer-specific effects, etc. Further study will be required to extrapolate on and update traditional safety thresholds of stimulation; however, the data presented are an important step forward in understanding the effects of intracortical stimulation and device design on the function and viability of surrounding brain tissue.

## Data Availability Statement

The datasets presented in this study can be found in online repositories. The names of the repository/repositories and accession number(s) can be found below: https://www.ncbi.nlm.nih.gov/, accession GSE202425.

## Ethics Statement

The animal study was reviewed and approved by Animal Care and Use Committee at Michigan State University and Animal Care and Use Committee at University of Michigan.

## Author Contributions

QW, BK, and MC: data collection. QW, BK, and BE: data analysis. QW, BK, EP, and JW: study design and planning and manuscript preparation. EP and JW: project oversite and management. All authors contributed to the article and approved the submitted version.

## Conflict of Interest

JW has a financial interest in Epic Medical, Inc., which is commercializing electrodeposited platinum iridium. The remaining authors declare that the research was conducted in the absence of any commercial or financial relationships that could be construed as a potential conflict of interest.

## Publisher’s Note

All claims expressed in this article are solely those of the authors and do not necessarily represent those of their affiliated organizations, or those of the publisher, the editors and the reviewers. Any product that may be evaluated in this article, or claim that may be made by its manufacturer, is not guaranteed or endorsed by the publisher.

## Supplementary Material

Supplementary Figure 1 Spatial expression of genes of interest in the chronic experiments. **(A)** *Cxcl13*, **(B)** *C3*, **(C)** *Junb*, **(D)** *Arc* expression at increasing distances from the expected location of the electrode tract in each sample.
