# Supplementary figures and images for: Spatial Transcriptomics as a Novel Approach to Redefine Electrical Stimulation Safety
Source: Front Neurosci. 2022 Jul 19;16:937923. doi: 10.3389/fnins.2022.937923 (PMC9344921; doi:10.3389/fnins.2022.937923)

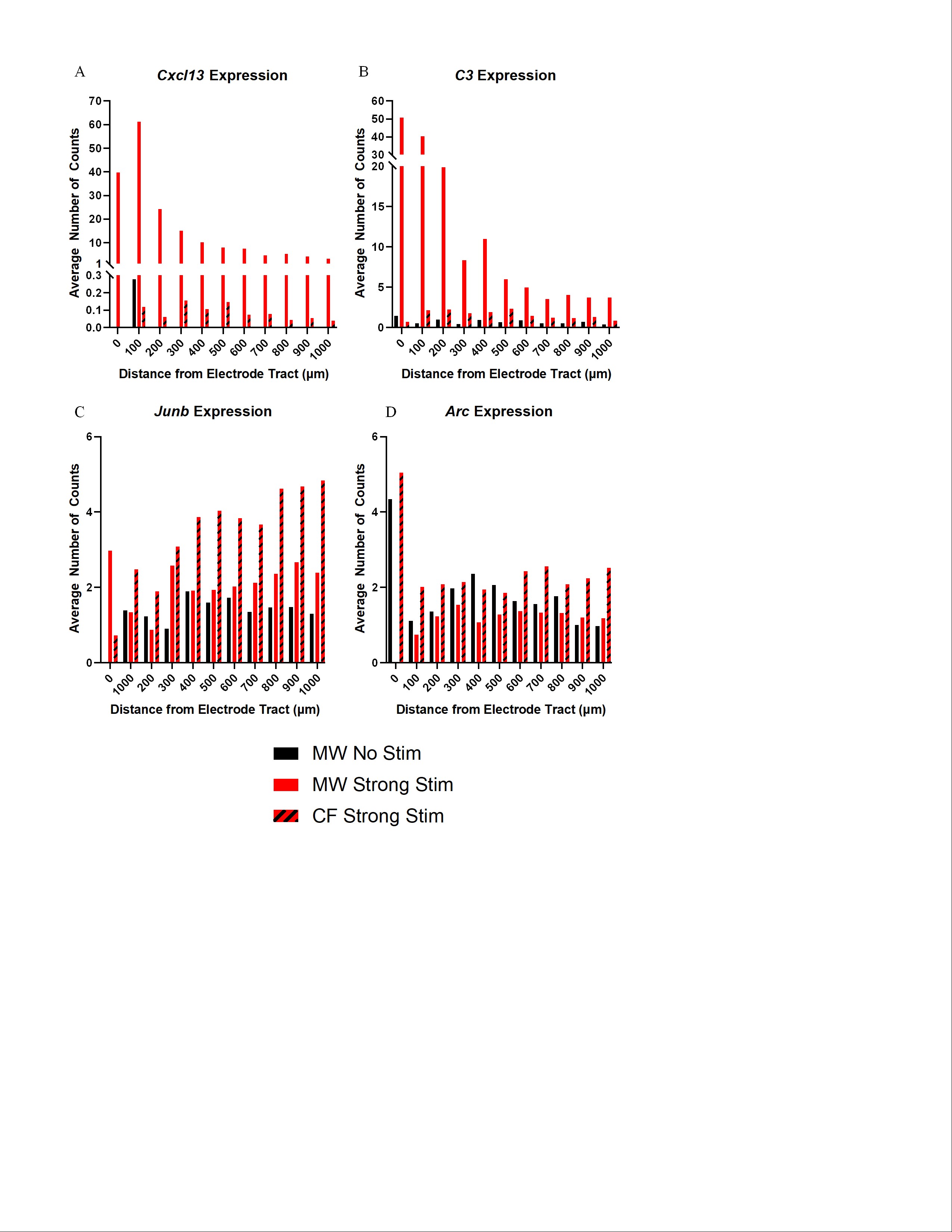

Supplement: Supplementary Figure 1 — Spatial expression of genes of interest in the chronic experiments. (A) Cxcl13, (B) C3, (C) Junb, (D) Arc expression at increasing distances from the expected location of the electrode tract in each sample. [file Image_1.JPEG]
